# Investigating the Effects of Lithium Phosphorous Oxynitride Coating on Blended Solid Polymer Electrolytes

**DOI:** 10.1021/acsami.0c09113

**Published:** 2020-08-13

**Authors:** Jed LaCoste, Zhifei Li, Yun Xu, Zizhou He, Drew Matherne, Andriy Zakutayev, Ling Fei

**Affiliations:** † National Renewable Energy Laboratory, Materials Science Center, Golden, Colorado 80401, United States; ‡ Department of Chemical Engineering, Institute for Materials Research and Innovation, University of Louisiana Lafayette, Lafayette, Louisiana 70504, United States

**Keywords:** LiPON, solid-state, hybrid bilayer electrolyte, molecular weight, critical thickness, lithium-ion batteries

## Abstract

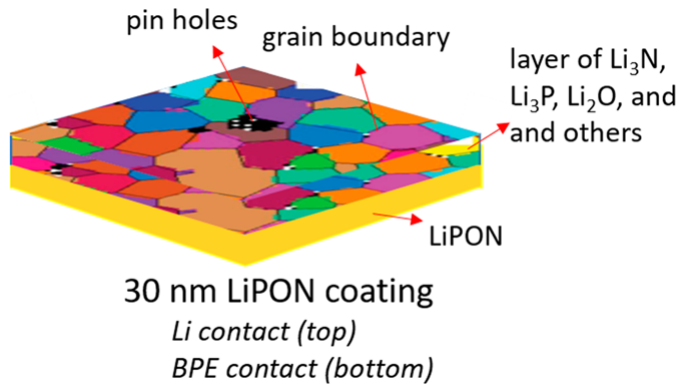

Solid-state electrolytes are very promising to enhance the safety of lithium-ion batteries. Two classes of solid electrolytes, polymer and ceramic, can be combined to yield a hybrid electrolyte that can synergistically combine the properties of both materials. Chemical stability, thermal stability, and high mechanical modulus of ceramic electrolytes against dendrite penetration can be combined with the flexibility and ease of processing of polymer electrolytes. By coating a polymer electrolyte with a ceramic electrolyte, the stability of the solid electrolyte is expected to improve against lithium metal, and the ionic conductivity could remain close to the value of the original polymer electrolyte, as long as an appropriate thickness of the ceramic electrolyte is applied. Here, we report a bilayered lithium-ion conducting hybrid solid electrolyte consisting of a blended polymer electrolyte (BPE) coated with a thin layer of the inorganic solid electrolyte lithium phosphorous oxynitride (LiPON). The hybrid system was thoroughly studied. First, we investigated the influence of the polymer chain length and lithium salt ratio on the ionic conductivity of the BPE based on poly(ethylene oxide) (PEO) and poly(propylene carbonate) (PPC) with the salt lithium bis(trifluoromethanesulfonyl)imide (LiTFSI). The optimized BPE consisted of 100 k molecular weight PEO, 50 k molecular weight PPC, and 25(w/w)% LiTFSI, (denoted as PEO100PPC50LiTFSI25), which exhibited an ionic conductivity of 2.11 × 10^–5^ S/cm, and the ionic conductivity showed no thermal memory effects as the PEO crystallites were well disrupted by PPC and LiTFSI. Second, the effects of LiPON coating on the BPE were evaluated as a function of thickness down to 20 nm. The resulting bilayer structure showed an increase in the voltage window from 5.2 to 5.5 V (vs Li/Li+) and thermal activation energies that approached the activation energy of the BPE when thinner LiPON layers were used, resulting in similar ionic conductivities for 30 nm LiPON coatings on PEO100PPC50LiTFSI25. Coating BPEs with a thin layer of LiPON is shown to be an effective strategy to improve the long-term stability against lithium.

## 1 Introduction

Conventional lithium-ion batteries typically use highly flammable organic solvents, such as diethyl carbonate, as a transport medium for lithium ions. Potential risks arise because of these solvents including leakage, fire, volatilization, and even explosions.^1−10^ To accommodate these issues, battery manufacturers must use bulky casing and other stringent safety features, resulting in batteries that have reduced energy density and higher levels of waste material.^3^ Furthermore, these safety features still do not entirely prevent the risks associated with organic liquid electrolytes. Solid-state electrolytes (SSEs) are very promising for the development of safer lithium batteries by focusing on the elimination/substation tiers on Occupational Safety and Health Administration ’s hierarchy of controls. As SSEs are in the solid state, there is a much lower chance of leakage, fire, and explosions. In addition, solid electrolytes also act as a separator material, and lithium metal can be used directly as the anode, allowing for more compact designs to be achieved, resulting in cells that offer high energy and power densities.^1^ Furthermore, solid-state ion conductors play a pivotal role in the development of new generations of technology in other fields such as electrochromic devices, sensors, and fuel cells.^11−13^

SSEs are divided into three major classes, solid polymer electrolytes (SPEs), inorganic solid electrolytes (ISEs), and hybrid solid electrolytes (HSEs). SPEs offer excellent physical properties for use in flexible battery technology and wearable electronics.^6^ However, because of the poor mechanical and chemical properties of SPEs, minimal protection from dendrite formation is offered, and the polymer may be subjected to reactive and degradable interfaces between lithium metal and the SPE.^2,6^ ISEs, on the other hand, offer complementary properties to SPEs. For instance, ceramic materials have been shown to be more stable and robust against lithium metal and lithium dendrites; however, their brittle nature causes mechanical failure when not supported by a ductile material, especially for thin film ceramics.^14^ HSEs can be engineered to synergistically combine the advantages of both SPEs and ISEs.

Poly(ethylene oxide) (PEO), a semicrystalline polymer, is excellent at solvating high concentrations of ionic salts and offers relatively high ionic conductivity at room temperature.^13^ However, PEO-based electrolytes can form crystal complexes with lithium salts, especially for high-molecular-weight systems where recrystallization is thermodynamically favored.^15^ The formation of these crystalline complexes results in a polymer electrolyte that has a dependence on thermal history because of recrystallization kinetics. To further decrease the effects of this phenomenon, it has been shown that blending PEO with an amorphous polymer is an effective strategy to mitigate this issue.^16^ Amorphous polymer electrolytes offer better lithium transport properties than their crystalline counterparts.^1,3,6,13,16−19^ Higher ionic conductivity in amorphous phases is often explained through dynamic bond percolation theory, where local segmental motion of the polymer chains, combined with an applied electric field, causes lithium ions to hop between coordination sites along the polymer chains.^1^ Poly(propylene carbonate) (PPC), an amorphous biodegradable copolymer of carbon dioxide and propylene oxide, has received a lot of attention recently because of the structure containing similar repeat units to carbonate-based liquid electrolytes.^20^ The shortage of PPC is its poor mechanical properties. Blending PEO with amorphous PPC is an effective strategy to reduce the crystallinity of the PEO as well to enhance the mechanical properties of PPC. In blended polymer electrolytes (BPEs), the blend ratio, polymer chain length, and lithium salt concentration all play critical roles in the ionic conductivity. Although two independent research groups have demonstrated that the 1/1 weight blending ratio in the PEO/PPC system is optimal, there is still a lack of fair evaluation of the impact of lithium salt concentration and chain length of PEO and PPC under the same conditions or by the same research team.^16,19−22^ It will be of great significance to initiate a study on the lithium salt ratio and polymer chain length of PEO and PPC in the same system on the basis of the 1/1 blend ratio, which can offer comprehensive and unbiased understanding on the BPE system. Furthermore, it was found that the TFSI^–^ group of LiTFSI offers reduced mobility in polymer electrolytes along with the ability to partially plasticize PEO because of the bulky structure of the anion and formation of lithium complexes with the ether groups.^17^ Therefore, LiTFSI is selected in this study and so far, no incremental ratio study of this salt is reported for the selected system.

To further improve interfacial stability and the chemical resistance of a BPE to lithium dendrites, it is proposed that a thin coating of a ceramic electrolyte may be an excellent solution to this issue. Lithium phosphorous oxynitride (LiPON), a well-known ceramic material for thin film batteries, is a promising candidate to act as an interface stabilizer for polymer electrolytes. The mechanical properties of LiPON evaluated through nanoindentation show that LiPON exhibits a shear modulus of 31 GPa, a value over nine times that of lithium metal.^14,23^ It has been shown that LiPON fabricated onto a polymer electrolyte shows negligible interfacial resistance, whereas when the polymer electrolyte is cast on LiPON, there is an influence on ionic conductivity because of interfacial resistance.^14^ These results indicate that LiPON can act as a potential protective layer on an SPE without offering any significant interfacial resistance when fabricated on a polymer surface. The thickness of the LiPON layer is expected to influence ionic conductivity of the layered hybrid electrolyte system. However, it remains unclear how LiPON’s thickness influences the ionic conductivity because in the previous study the LiPON layer thickness was held constant. Evaluating the critical thicknesses is important for understanding the transport properties of lithium ions across the polymer/ceramic interface.

Herein, we report a PEO-PPC-LiTFSI/LiPON bilayer hybrid SSE consisting of a salt-in-blended-polymer electrolyte coated with the amorphous lithium conducting glass LiPON. First, the effects of LiTFSI mass loadings and polymer molecular weight in 1:1 PEO/PPC blends are investigated systematically. From there, the best three polymer electrolytes are determined by impedance spectroscopy. Impedance measurements are then taken over a range of temperatures to understand the effects of the polymer chain length on activation energy. X-ray diffraction (XRD) and optical microscopy are employed to understand the structure–property relationship for the blended electrolytes. Second, the best SPE is coated with different thicknesses of LiPON. Stability against lithium, voltage window, and temperature-dependent ionic conductivity are studied to understand the effect of LiPON coatings on the BPE. These experiments are performed as a function of LiPON thickness, to identify the critical LiPON thicknesses for protecting the BPE. The minimum LiPON thickness for protection of the polymer electrolyte against lithium is found to be 30 nm, indicating a promising method to improve the stability of the electrolyte film against the harsh lithium environment. For higher thicknesses, LiPON’s ionic conductivity dominates the transport of lithium ions, and for lower thicknesses, LiPON does not protect the BPE system from interfacial degradation.

## 2 Experimental Section

### 2.1 Chemicals

Poly(ethylene oxide) [PEO], poly(propylene carbonate) [PPC], lithium (bistrifluoromethanesulfonyl)imide [LiTFSI] (reagent grade), and anhydrous acetonitrile (HPLC grade) were purchased from Sigma-Aldrich Corporation (St. Louis, Mo). A 2″ lithium phosphate target (99.99%) was purchased from Plasmaterials Inc. Before use, all polymers were dried under vacuum at 60 °C for 24 h, and LiTFSI was dried at 100 °C for 24 h; all materials were then stored in a dry box.

### 2.2 Polymer Electrolyte Preparation

First, PPC and PEO were blended in equal mass ratios and then mixed with different mass loadings of LiTFSI ranging from 20 to 40%. The solid materials were then dissolved in anhydrous acetonitrile and stirred overnight. To prepare the films, a simple and inexpensive solution casting method was used. Electrolyte precursor solutions were cast onto various materials for different test procedures and then dried in a vacuum oven at 60 °C overnight. Throughout this report, the abbreviation PEOxPPCyLiTFSIz is used to identify samples, where *x* = 100, 300, and 600 and *y* = 50 and 232 denote the average molecular weight of the polymers divided by 1000, and *z* is the mass percentage of LiTFSI in the electrolyte blend.

### 2.3 LIPON Synthesis

Lithium phosphorous oxynitride was synthesized by radio frequency magnetron sputtering using a lithium phosphate target. The glovebox conditions were lower than 5 ppm water and oxygen. During the deposition, a power density of 25.8 W·in^–2^ was applied and a base pressure of 3 × 10^–8^ Torr was obtained. To generate plasma, argon (99.999% purity) was introduced at a constant pressure of 20 mTorr; nitrogen (99.999% purity) was then introduced at a 1:1 ratio, and the pressure was reduced to 3 mTorr. The distance between the target and the substrate is 8 cm, and the target is placed parallel to the substrate. The chamber temperature is around room temperature. The deposition rate of LiPON is determined by measuring the thickness of the film using a Dektak profilometer on a film grown for a set time. The growth rate is assumed linear with time. The substrate is changed depending on the test performed. For investigating the interaction between LiPON and the polymer electrolyte, the polymer electrolyte samples are cast onto blocking electrodes and then coated with different thicknesses of LiPON.

### 2.4 Characterization

Fabricated polymer electrolytes were observed through confocal microscopy (inVia confocal Raman microscope, Renishaw, Gloucestershire, UK) and XRD (XRD, Rigaku DMAX 2500, The Woodlands, Texas, USA). XRD patterns were obtained over a 2Θ range of 15 to 70° with CuKα radiation at room temperature with a scan rate of 3°/min. Differential scanning calorimetry (DSC) was used to assess the changes in crystallization behavior for the best electrolyte system compared to pure PEO. DSC was performed on a PerkinElmer DSC 4000 differential scanning calorimeter over the range of −40 to 150 °C with a heating and cooling rate of 10 °C/min.

**Figure 1:**
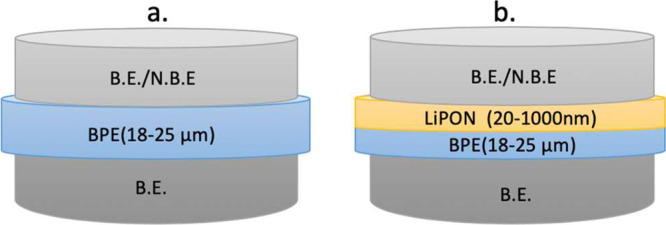
Cell arrangements for testing: (a) BPEs and (b) bilayer structured hybrid electrolytes. Arrangements shown for both blocking (B.E.) and nonblocking electrodes (N.B.E).

Ionic conductivity of the SPE and HSEs was determined through impedance spectroscopy using a Bio-Logic SA Potentiostat/Galvanostat (France) on a 2032 coin-cell with electrolyte films sandwiched between two blocking electrodes, shown in Figure 1. The frequency range used was from 1 MHz to 100 mHz with an amplitude of 5 mV. Temperature’s influence on ionic conductivity was assessed for the best three polymer electrolytes and LiPON-coated optimized electrolyte by placing the test cells in an oven. The oven was set to the desired temperature, and then after the temperature was reached, the cells were rested for 30 min to ensure that the temperature of the cell is the same as the temperature in the oven.

To investigate the effects of thermal history, the samples were heated and cooled twice. Linear sweep voltammetry (LSV) is the technique used to assess the electrolyte’s anodic stability against lithium. To determine the influence of contact time on charge transfer resistance and interfacial stability with lithium metal, electrochemical impedance spectroscopy (EIS) with the same parameters for ionic conductivity was used. To assess polarization influence, EIS spectra were measured before and after the electrolyte being polarized at 2 V versus the open circuit potential for 20 min. The configuration of the electrolyte and cell measurement is shown in Figure 1.

## 3 Results and Discussion

**Figure 2:**
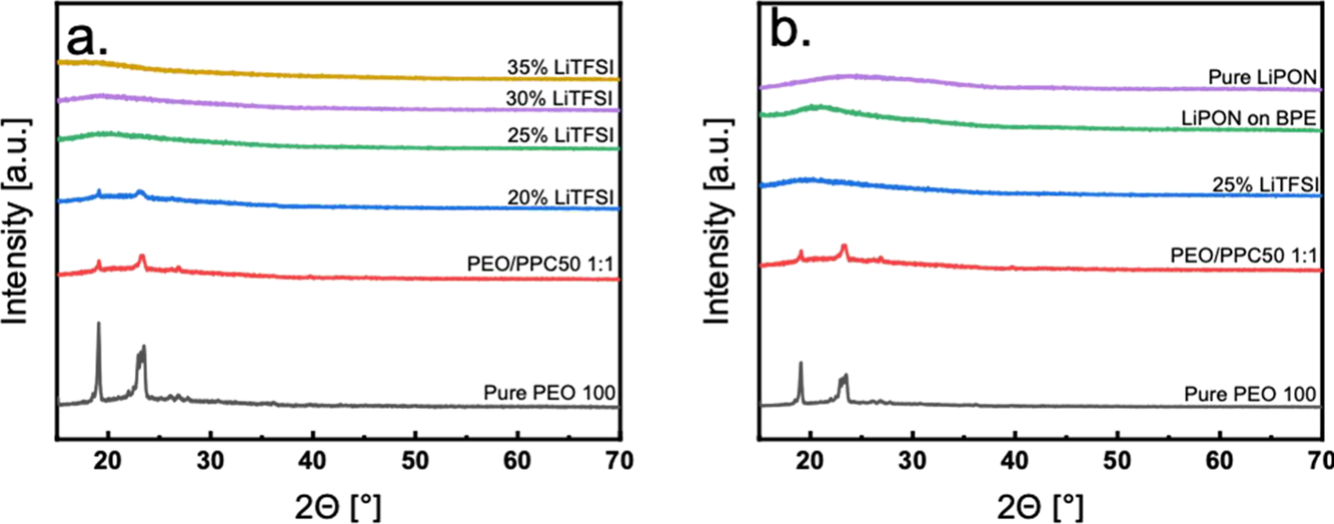
Typical XRD patterns for (a) PEO100, PEO100PPC50 blend, and the resulting blend electrolyte films with different salt ratios, and (b) optimal BPE (PEO100PPC50LiTFSI25), pure LiPON, and LiPON coated BPE.

XRD is used to identify the crystalline phases and examine the crystallinity of the electrolyte systems. As shown in Figure 2 and Figure S1, all molecular-weight level PEO samples have characteristic 2Θ peaks at around 19 and 23°. This confirms that the pure PEO is a semicrystalline polymer, and the peak reductions are observed because of blending with the amorphous PPC and complex formation with lithium ions. As found in Figure 2a, with the addition of equal mass of PPC to PEO, a large drop in peak intensity can be seen. This indicates that the PEO’s crystalline structures are being disrupted by the amorphous PPC. Incorporating LiTFSI allows the bulky TFSI^–^ groups to further reduce the crystallinity, as evidenced by the broadening of the peaks and the reduction in peak intensities, and the peak completely disappears at higher LiTFSI concentrations. This is a result of the formation of lithium complexes with the polar groups in both PEO and PPC. The XRD patterns for higher molecular-weight PEO systems shown in Figure S1a,b, PEO300 and PEO600, respectively, demonstrate very similar results to those of the lower molecular-weight PEO system with the low molecular-weight PPC (50 k). Figure S1c shows the XRD pattern for the higher molecular-weight PPC system. The weak peaks at 19 and 23° indicate that the higher molecular-weight PPC disturbs the PEO crystalline phases effectively. However, there is substantial phase separation seen in microscopy images shown in the following section, indicating the poor blending between high molecular-weight PEO and PPC. Figure 2b shows the XRD patterns for the PEO100PPC50 systems, LiPON, and the PEO100PPC50LiTFSI25 coated with a layer of LiPON. This measurement confirms that the LiPON is amorphous and that no new crystalline peaks form when LiPON is deposited on PEO100PPC50LiTFSI25.

**Figure 3:**
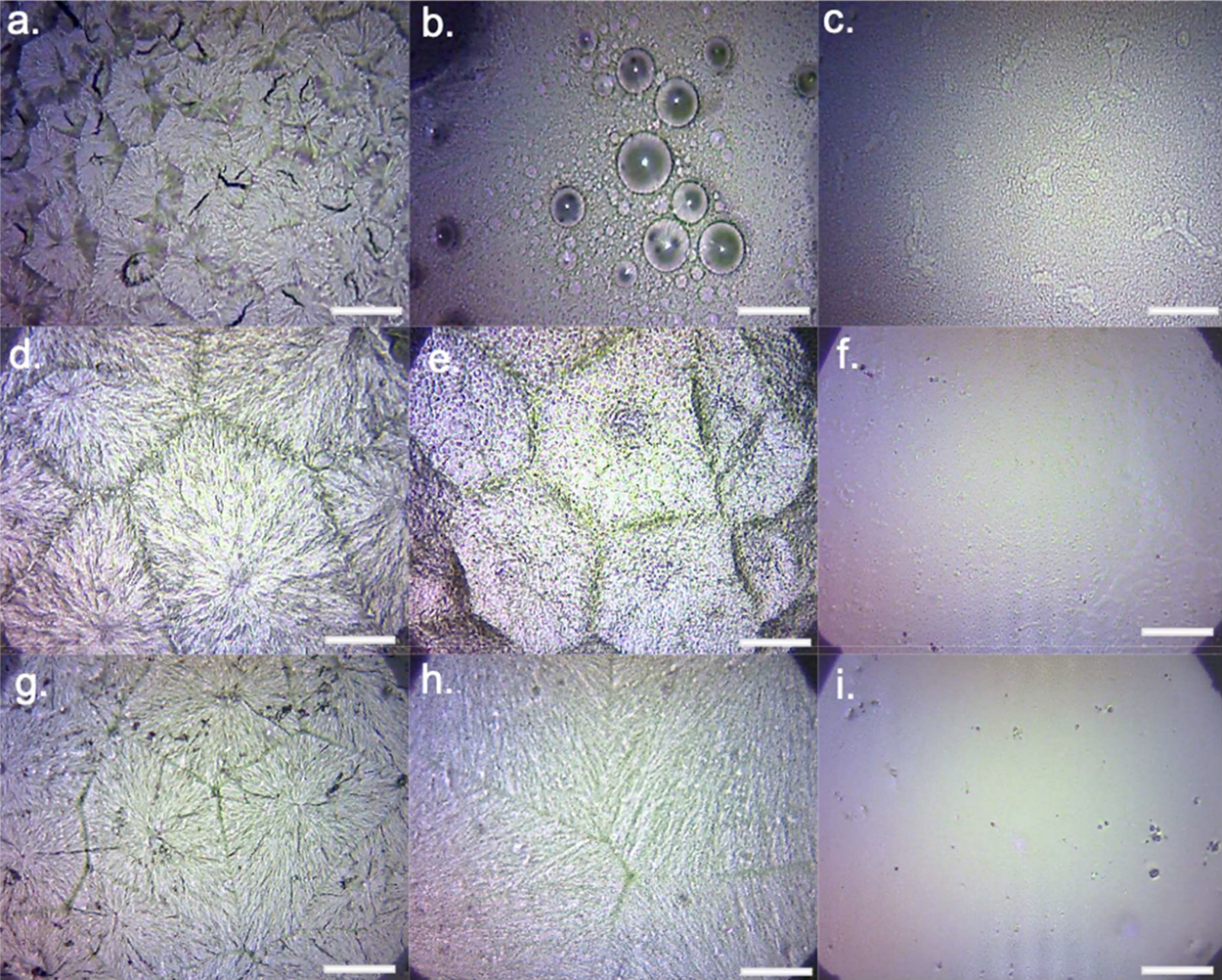
Optical microscopy images of (a) PEO100, (b) PEO100PPC50 (1:1), (c) PEO100PPC50LiTFSI25, (d) PEO300, (e) PEO300PPC50 (1:1), (f) PEO300PPC50LiTFSI25, (g) PEO600, (h) PEO300PPC50 (1:1), and (i) PEO300PPC50LiTFSI25 [Magnification 20x/ Scale Bar: 50 μm].

Optical microscopy images of the polymer electrolyte samples before coating with LiPON were taken to observe the change in the microstructure in the PEO/PPC blended electrolyte system. Figure 3 shows the pure PEO (a, d, and g), the PEO/PPC blends (b, e, and h), and the optimized PEOxPPC50LiTFSI systems. The optimized systems were determined through impedance measurements and are supported by the reduction in crystallinity as determined by XRD. The pure PEO samples show a well-defined crystalline structure as observed through microscopy images. For the lower molecular-weight samples, the PEO crystals are much smaller than PEO300 and PEO600. With the addition of PPC, it can be seen from Figure 3b that for the low molecular-weight PEO electrolyte films, PPC disrupts the crystalline phases of the PEO resulting in a distribution of amorphous PPC-rich areas and smaller spherules of PEO-rich regions. However, with the increase in PEO’s molecular weight, the disruption of the crystallinity is less intense as demonstrated in Figure 3e,h and evidenced by the well-defined crystal structures. Introducing the LiTFSI salt appears to completely homogenize the electrolyte system for all cases (Figure 3c,f,i). The results of these measurements indicate that the blending of PEO and PPC and introducing LiTFSI salt is an effective strategy to reduce the crystallinity of the samples. Figure S2 shows the blending samples of higher molecular-weight PPC with the lower molecular-weight PEO. When the PPC’s molecular weight was increased, and the lower molecular weight PEO was used, phase separation and film inconsistencies were observed even after the addition of 30 w/w % LiTFSI. The disruption of the crystallinity of the lower molecular-weight PEO/PPC systems is due to lithium-ion complex formation between the polar groups in the PPC and PEO chains. With larger PPC chains, the degree of chain folding and entanglement is severe, and the lithium-ion complex interaction is not strong enough to unfold or unknot the chain. Two distinct phases are observed through microscopy, indicating poor miscibility in the higher molecular-weight polymer system. Flory–Huggins’ solution theory is also used to evaluate the PEO100PPCx system miscibility and compare with experimental results. The calculation result is plotted as shown in Figure S3. It can be seen that the change in free energy of mixing increases with increasing PPC molecular-weight, indicating that higher molecular-weight leads to higher degrees of immiscibility. The trend from the calculation agrees well with the experimental result that in high-molecular-weight samples, extreme phase separation is noticed even with the addition of LiTFSI. The detailed calculation process is presented right after Figure S3 in the Supporting Information.

**Figure 4:**
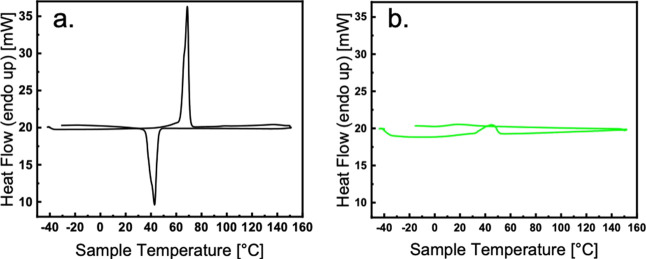
DSC measurements for (a) PEO100 and (b) PEO100PPC50LiTFSI25-optimized BPE.

To assess the thermal behavior of the optimized electrolyte system, DSC is performed on pure PEO100 and PEO100PPC50LiTFSI25. Figure 4 shows the results of this experiment; the melting temperature of pure PEO100 is found to be at 69 °C as seen in Figure 4a. Additionally, the recrystallization temperature is found to be centered at 43 °C. Upon the addition of PPC50 and 25% w/w LiTFSI, significant changes in the DSC curve are noticed. Figure 4b shows a melting temperature around 45 °C for the blended system, and no recrystallization is observed. These results confirm that the addition of PPC50 and LiTFSI25 disrupts the recrystallization behavior of the electrolyte system. Because the material does not exhibit any obvious recrystallization, the bulk of the material is amorphous at room temperature, allowing for optimal ionic conductivity in the system.

**Figure 5:**
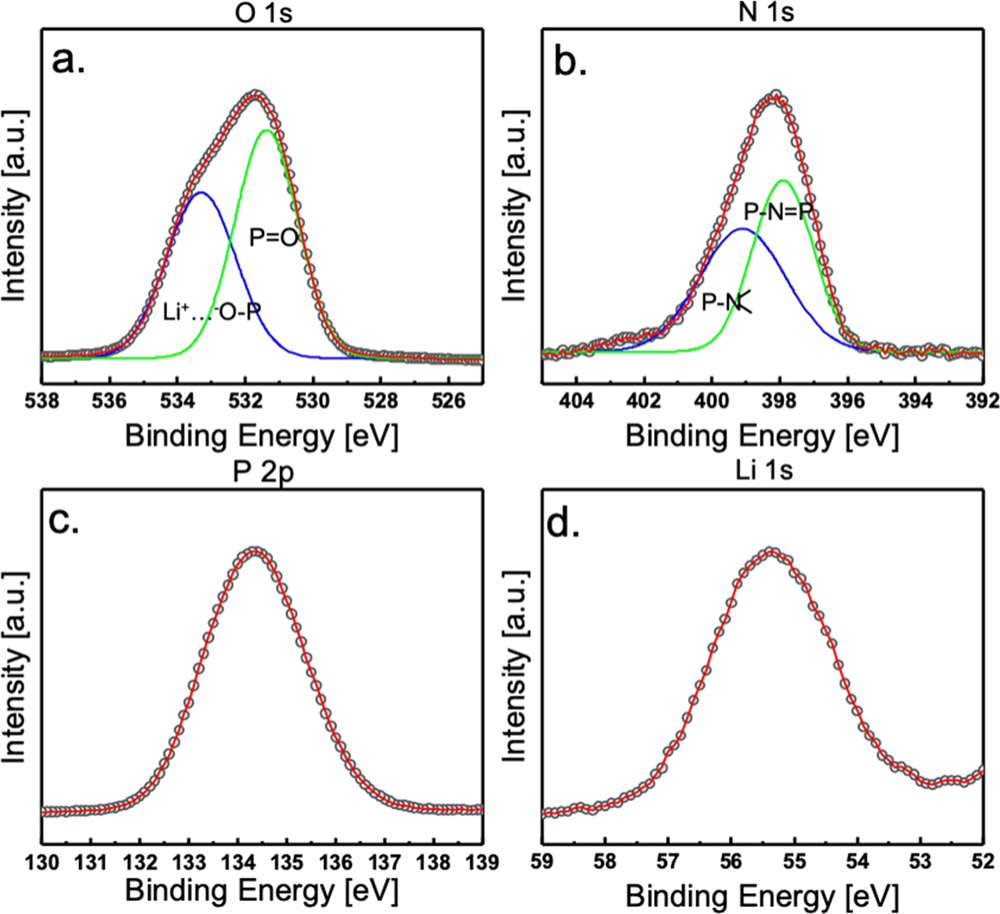
X-ray photoelectron spectroscopy (XPS) spectrum for LiPON showing peaks in descending binding energy for (a) O 1s, (b) N 1s, (c) P 2p, and (d) Li 1s.

After gaining insight into the BPE system, the electrolyte was then coated with thin layers of LiPON for further study. The confirmation of LiPON synthesis and its composition were verified by XPS. The XPS spectrum in Figure 5 displays nitrogen’s 1s (N1s) peaks at binding energies of 399.5 and 398 eV, corresponding to nitrogen with triple coordination (NT) and double coordination (ND), respectively.^24,25^ The XPS data indicate that the primary nitrogen configuration is doubly coordinated as determined by the peak shape being shifted toward 398 eV. The binding energy difference between ND and NT is calculated to be 1.5 eV; similar values are reported by Wang and Schwöbel’s groups.^26,27^ The oxygen 1s (O1s) peak exhibits an asymmetry, corresponding to the P–O–P bridging oxygens and the P–O–Li and P=O nonbridging oxygens at the binding energies of 534.5 and 531 eV, respectively.^24−27^ Evaluation of the Li1s and P2p signals was performed with single profile fits because of the symmetry of the peaks. The Li1s peak, occurring at a binding energy of 55.3 eV, indicates that the presence is only one containing lithium species contributing to the XPS peak.^27^ It is also noteworthy that the profile of the lithium spectrum seems not to be perfectly symmetrical. This could be due to the impact of the N triple bond and double bond on the LiPON structure, shown in Figure S6. As nitrogen has a higher electronegativity than P (N electronegativity is 3.0 and 2.1 for P), the NT and ND will induce different electron distribution of the adjacent P, which then slightly influences the Li environment, leading to some asymmetry in XPS measurements. The feature of our Li1s is similar to the results from several other reported LiPON studies where small asymmetry was observed.^28−31^ It has also been found that the ND/NT ratio directly affects the Li ion conductivity of LiPON, also suggesting the slightly different local environment of Li in LiPON.^32,25^ The P2p peak around 133 eV corresponds to the presence of the phosphate structure; the symmetry of this peak indicates that there is no other structure of phosphorous such as phosphide.^27^

**Figure 6:**
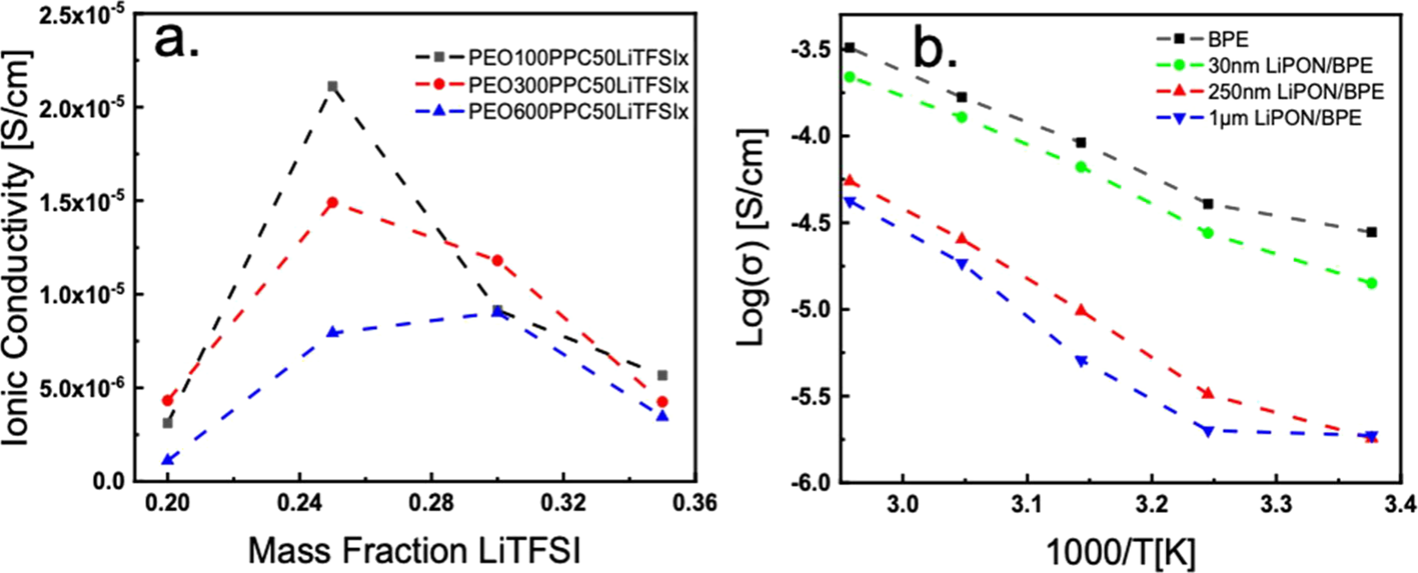
Ionic conductivity of each polymer electrolyte system as a function of mass fraction of LiTFSI (a) PEO (100, 300, and 600) PPC50LiTFSIx and (b) temperature effect on PEO100PPC50LiTFSI25 (BPE) and LiPON-coated BPE’s ionic conductivity.

Impedance spectroscopy, shown in Figure S7, is used to probe the ionic conductivity of the electrolytes. From EIS measurements, the ionic conductivities of the polymer electrolytes were determined using the bulk resistivity, *R*_b_ (Ω). To calculate the value of the ionic conductivity (σ) in S/cm, the following equation is used:
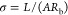
where *L* (cm) is the thickness of the electrolyte and *A* (cm^2^) is the cross-sectional area between the two blocking electrodes. The ionic conductivities were then plotted against the mass fraction of LiTFSI to determine the optimal salt concentration for each electrolyte. Findings from our study in Figure 6a show that the optimal LiTFSI salt loading giving is ∼25% for blended electrolyte systems of PEO and PPC. It agrees well with the literature results that the optimal LiTFSI loading is between 20 and 30% for PEO systems.^33^ After the optimal point is reached, the electrolyte sees a dramatic decrease in ionic conductivity; this phenomenon occurs because of ion agglomeration.^34^ The system with the best ionic conductivity is PEO100PPC50LITFSI25. It is also observed that for higher molecular-weight polymers, more LiTFSI is required to reach the optimal ionic conductivity for that sample set. Furthermore, higher molecular-weight polymers offer lower ionic conductivities because of larger chains offering lower mobility. Between the polymer samples, it appears that the influence of PPC’s molecular weight is very substantial as shown in Figure S8a. Ionic conductivity values for substituting PEO100 for PEO600 result in a 57.3% drop from the optimized systems, and PPC shows a 93.4% reduction in ionic conductivity from substituting PPC50 to PPC232. Because of the clear negative impact of larger molecular-weight PPC, PPC50 is selected for continuous study.

Figure S8b shows the polymer electrolytes’ ionic conductivity as a function of temperature. From these data, an Arrhenius-type relationship is used to evaluate the activation energies for the different molecular-weight PEO samples and is shown in Table 1. Comparing activation energies for the different molecular-weight samples, the longer chain PEO electrolytes have higher activation energies. For the PEO100PPC50LiTFSI25 electrolyte, the activation energy is 21.8 kJ/mol. For the higher molecular-weight systems, the activation energies are the higher values of 22.5 and 26.5 kJ/mol for the PEO300PPC50- and PEO600PPC50-optimized systems, respectively. This can be attributed to the larger chains having lower mobility because of chain entanglement, resulting in higher energy required for lithium ions to hop between coordination sites. Because the lower molecular-weight polymer has shorter chains, the local segmental motion of the polymer chains offers better ion mobility. The lower molecular-weight polymer electrolyte, PEO100PPC50LiTFSI25, has both the lowest activation energy and the highest ionic conductivity at room temperature.

**Table 1:** Activation Energies and Ionic Conductivity Values for the Best Three Polymer Systems and 30 nm, 250 nm, and 1 μm LiPON-Coated BPE

sample	activation energy (kJ/mol)	ionic conductivity at 23 °C (S/cm)
PEO100PPC50LiTFS125 (BPE)	21.8	2.11E × 10^–5^
PEO300PPC50LiTFS125	22.5	1.49E × 10^–5^
PEO600PPC50LiTFS130	26.5	9.02E × 10^–6^
30 nm LiPON on SPE	24.4	1.41E × 10^–5^
250 nm LiPON on SPE	31.1	1.81E × 10^–6^
1 μm LiPON on SPE	35.9	1.87E × 10^–6^

Figure 6b shows the temperature dependence of LiPON-coated PEO100PPC50LiTFSI. It is found that depositing LiPON on the surface of the highest ionic conductivity sample, PEO100PPC50LiTFSI25, results in an increase in activation energy and a decrease in ionic conductivity. This increase in activation energy is due to the activation energy of the LiPON being higher than that of the base polymer electrolyte.^25,35^ With thicker LiPON on the surface, the activation energy becomes higher. As a result, 30 nm of LiPON coating shows a value close to the BPE, while samples with 250 nm and 1 μm LiPON coating have lower ionic conductivity (Figure 6b).

A set of temperature-dependent measurements on the thermal history effect on SPE samples are also taken and shown in Figure S9. From these experiments, it is found that the lower molecular-weight PEO100PPC50LiTFSI25 electrolyte has a lower dependence on temperature than the higher molecular-weight PEO samples. These results are consistent with previous literature,;^16^ extending this work, we show that the ionic conductivity of the higher molecular-weight PEO300 and PEO600 samples initially is low compared to subsequent cooling then heating. After the initial heating, the polymer electrolytes appear to offer comparable ionic conductivities as the ionic conductivity data overlap well. From these data, it is concluded that the thermal history effects because of recrystallization are effectively eliminated in PEO100PPC50LiTFSI25.

**Figure 7:**
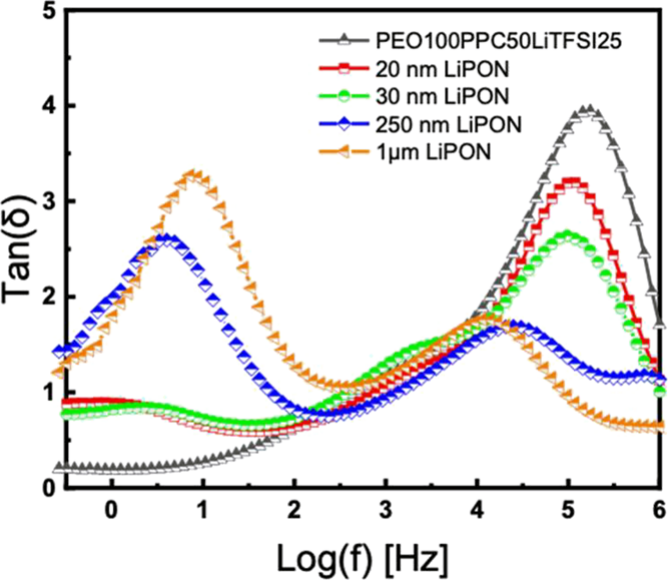
Loss tangent spectra for uncoated PEO100PPC50LiTFSI25 and different thickness LiPON-coated samples.

The loss tangent, tan(δ), is the ratio of the real impedance (Z’) to the imaginary impedance (Z”) and represents the ratio of lost energy to stored energy under an applied electric field.^36^ On a plot of tan(δ) versus the frequency, a maximum corresponds to the energy lost because of dipole relaxation. Higher frequencies correspond to a faster relaxation process and therefore faster lithium-ion transfer kinetics. In Figure 7, the polymer electrolyte, PEO100PPC50LiTFSI25, only shows one characteristic peak in the high frequency range. When a thin layer of LiPON is deposited on the surface of the polymer electrolyte, an additional peak arises. These correspond to the two phases in the electrolyte sample showing two different relaxation times for the polymer and the LiPON electrolyte. The peak in the lower frequency range is related to the dipole relaxation within the ceramic electrolyte, which is much slower compared to the polymeric electrolyte.^37^ With the thicker layers of LiPON, the two characteristic peaks become more evident because of the ion transport in the ceramic layer becoming more significant and dominant. It is shown that the thinner the LiPON coating, the more similarly it behaves to the BPE.

**Figure 8:**
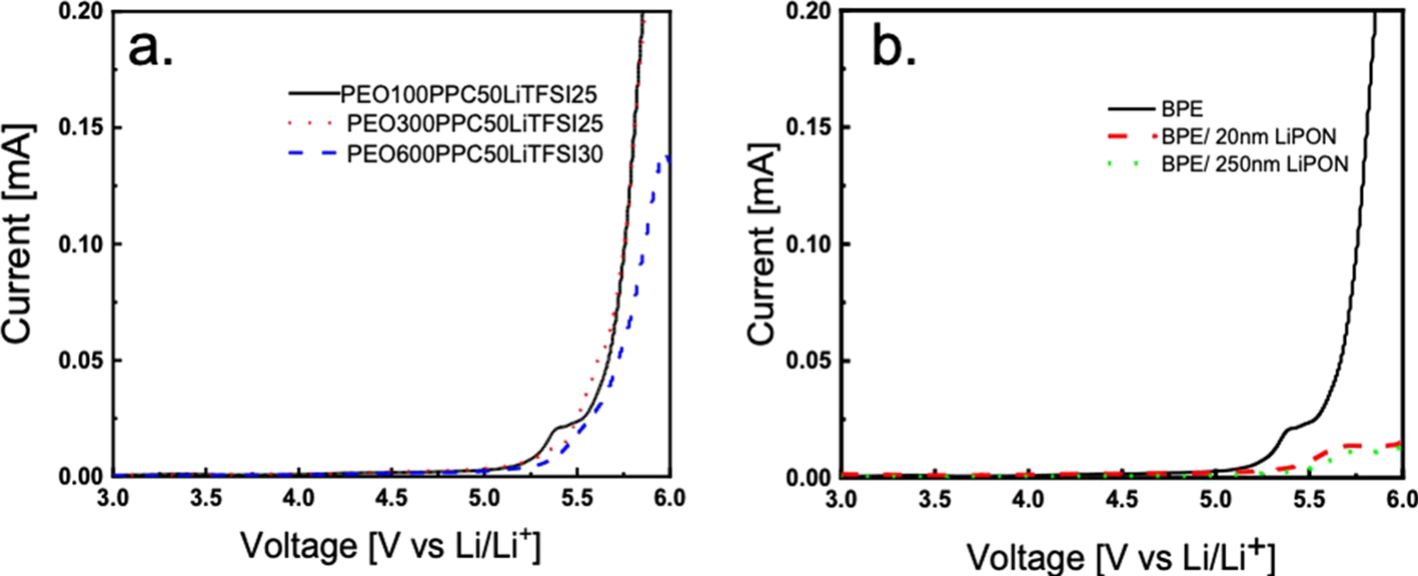
Linear sweep voltammograms of (a) PEOxPPC50LiTFSI-optimized systems and (b) PEO100PPC50LiTFSI25 (BPE) and LiPON.

Electrochemical stability is another important parameter to consider when selecting materials for lithium metal batteries. Having wide electrochemical windows allows for compatibility with a wider range of electrode materials. Developing electrolytes with wide electrochemical windows is important for the development of solid-state cells. The voltage window for the three best polymer electrolyte samples is determined by LSV. For the polymer electrolytes without any coating of LiPON, the voltage windows are up to around 5.25 V vs Li/Li + as shown in Figure 8a. Compared to literature values, PEO/LiTFSI has been shown to have a voltage window up to 5.3 V, and PPC-based electrolytes exhibit voltage windows up to 4.6 V.^6,21,38^ The value we observed in the blended system is very close to that of PEO/LiTFSI. With the addition of LiPON, the voltage window is further improved to reach a voltage of 5.5 V, Figure 8b, which corresponds to the voltage window of LiPON as reported in previous literature.^14^ This increase in the voltage window is attributed to LiPON acting as a protective layer between the lithium and the SPE.

**Figure 9:**
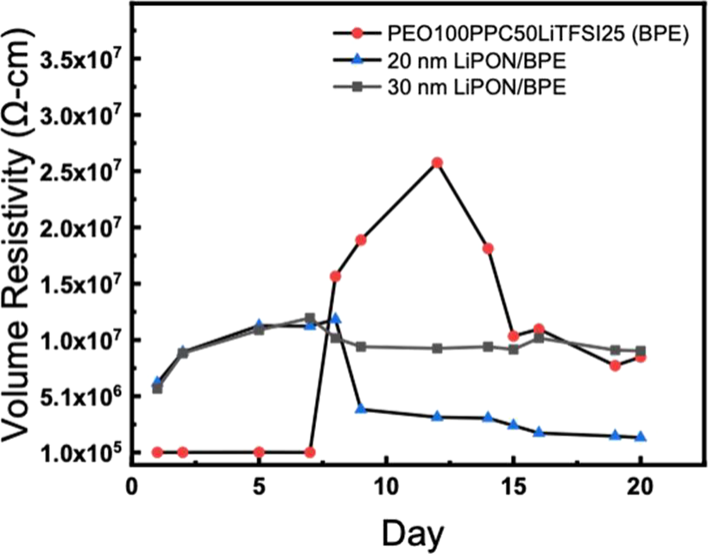
Bulk resistivity of the PEO100PPC50LiTFSI25 (BPE), BPE with 20 nm LiPON, and BPE with 30 nm LiPON to assess the effects of storage times of the electrolytes against lithium metal.

The evaluation of the time-dependent EIS measurements for the best BPE and its LiPON-coated derivatives is performed to assess the stability against lithium metal. Impedance measurements were run frequently and recorded, and changes in the impedance spectra are observed. As shown in Figure S10a,b, and c, there are significant differences in the impedance spectra over a period of 20 days for PEO100PPC50LiTFSI25 (BPE), 20 nm LiPON-coated BPE, and 30 nm LiPON-coated BPE. Higher thicknesses of LiPON are not considered for this study because of the poor ionic conductivity values. For the BPE against lithium, Figure 9 shows no significant changes in the resistivity until day 8, where the charge transfer resistance sees a sudden increase from low values of charge transfer resistance (order of 10^5^ Ω·cm) to 1.58 × 10^7^ Ω·cm. This indicates that the interface between the BPE and the lithium is unstable. It could be because the lithium metal interacts with the carbonate groups in poly(propylene carbonate) to form nonionically conductive parasitic products resulting in an increase in resistivity.^22^ As already mentioned, the obvious increase starts at day 8, and the interfacial reaction kept going until day 12 until the volume resistivity approached maximum. Afterward, the values go down, which could be due to the gradual decomposition of the previously formed parasitic products. Once there are new BPE surfaces exposed, the parasitic reaction will occur again, resulting in an increase of volume resistivity again and evidenced by the increasing trend in the figure starting from day 18. Therefore, the volume resistivity for the BPE system does not stabilize to a constant resistivity during the period of 20 days and may not be able to fully stabilize even given long enough times. Although the bare polymer electrolyte initially has low volume resistivity because of the excellent contact with lithium metal and high ionic conductivity, it as a standalone is not desirable for use because of the unstable interface between lithium and the electrolyte.

For all LiPON-coated samples, the bulk resistivity value on day 1 is around the same value of 6.15 × 10^6^ Ω·cm. The 20 nm LiPON-coated BPE shows very interesting behavior, and the bulk resistivity increases slowly, then drops after day 8, and slowly goes down to around 1.60 × 10^6^ Ω·cm. The reduction in volume resistivity is due to the formation of a passivation layer between LiPON and lithium. In the XPS study of this interface, it has been shown that the exposed LiPON reacts with lithium to produce a passivation layer of smaller units such as Li_3_P, Li_3_N, and Li_2_O. Further analysis indicates that the LiPON/lithium interface stabilizes after the full development of this layer, preventing further reactions.^27^ Some species formed (Li_3_N and Li_3_P) have ionic conductivities on the order of 10^–4^ and 10^–3^ S/cm, respectively, higher than LiPON’s value, offering a fast lithium-ion transfer path; therefore, we see a decrease trend of volume resistivity around day 8.^27,39,40^

**Figure 10:**
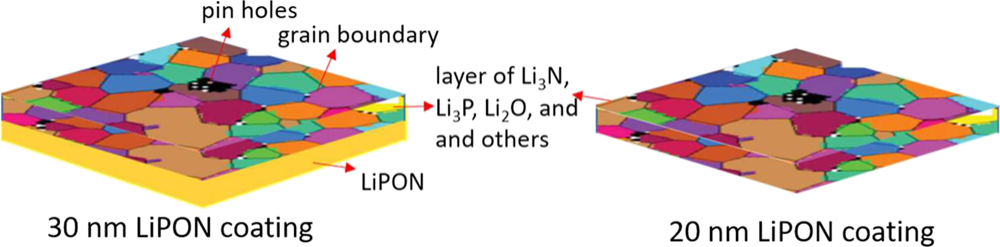
Schematic illustration of the likely interface formed at the LiPON/Lithium contact (Li contact at the top side, BPE at the bottom side).

The 30 nm sample shows almost the sample volume resistivity as the 20 nm sample in the initial 7 days, suggesting when the thickness of LiPON is below 30 nm, it does not have that much difference on ionic conductivity and the interfacial stabilization barely starts. Around day 9, the 30 nm LiPON-coated sample appears to be relatively stable with volume resistivity around 9.54 × 10^6^ Ω·cm during the rest period of testing days, different from the behavior of 20 nm LiPON-coated sample that shows a small decrease trend in resistivity gradually close to the original BPE value. This is likely because all 20 nm LiPON has been gradually consumed to form the passivation layer of higher ionic conductivity than LiPON. However in the case of 30 nm LiPON, it maystill have an intact LiPON layer beneath the passivation layer; therefore, the volume resistivity remains much more stable than the other two. A schematic illustration of the 30 and 20 nm LiPON samples after in contact with lithium is demonstrated in Figure 10. In terms of impact on volume resistivity, the sample with 20 nm LiPON coating is definitely more promising. However, the concern is the passivation layer with different species will have many grain boundaries and some pin holes formed, leaving it very vulnerable to dendrite puncturing through in a long run. LiPON coating thickness lower than 20 nm will raise the same concern and defeat the purpose of SSEs for improved safety. Taking into account the importance of stability and safety in electrochemical cells, 30 nm therefore is considered as the critical minimal coating thickness, as the intact LiPON layer underneath the passivation layer can resist dendrite piercing.

To assess the response to polarization, impedance spectra were taken before and after applying a potential of 2 V for 20 min. Figure S11 shows the change in the impedance spectra of the PEO100PPC50LiTFSI25 with and without a thin layer of LiPON (20 and 30 nm) after polarization of the sample for 20 min at 2 V against Li/Li^+^. The uncoated sample exhibits different EIS before and after polarization. This indicates a change in the diffusion kinetics of the uncoated samples, whereas for the LiPON-coated samples, EIS spectra do not change by much after polarization. Polarization data of this group of samples further indicate that the LiPON-coated samples have better stability at an applied voltage, which is desired for battery operation as the operation voltage will not remain constant.

## 4 Conclusions

We report a bilayered lithium-ion conducting HSE consisting of a BPE coated with a thin layer of the ISE lithium phosphorous oxynitride (LiPON). The effects of PEO and PPC’s molecular weights on BPE systems along with mass loading of LiTFSI on the ionic conductivity were systematically investigated. The highest ionic conductivity polymer electrolyte is achieved with the lower molecular-weight PEO and PPC. Lower molecular-weight polymers have more free volume and offer higher degrees of segmental motion resulting in higher ionic conductivities. It has also been shown that the higher degree of amorphous character results in higher ionic conductivity compared to the more crystalline electrolytes. We also found that coating the optimized BPE with an appropriate thickness of LiPON increases the voltage window and improves the stability of the electrolyte system with extended exposure to the lithium metal. Therefore, a thin layer of LiPON acts as a protective layer between the polymer electrolyte and lithium metal. The addition of LiPON to stabilize the electrolyte/lithium interface can be applied over a large range of polymeric electrolytes and has the potential to offer better lithium stability and offer comparable ionic conductivities to the base polymer electrolyte system.

## Supporting Information Available

XRD patterns of all the blends, optical microscopy images, calculated Gibb’s free energy, PPC’s molecular structure with color-coded functional groups, triple bond N and double bond N in LiPON, impedance spectra, ionic conductivity values, thermal history dependence, electrochemical impedance spectra, and functional group contributions for PPC and PEO (PDF)

## References

[ref1] Chen R.; Qu W.; Guo X.; Li L.; Wu F. The Pursuit of Solid-State Electrolytes for Lithium Batteries: From Comprehensive Insight to Emerging Horizons. Mater. Horiz. 2016, 3, 487–516.

[ref2] Chai J.; Liu Z.; Ma J.; Wang J.; Liu X.; Liu H.; Zhang J.; Cui G.; Chen L. In Situ Generation of Poly (Vinylene Carbonate) Based Solid Electrolyte with Interfacial Stability for LiCoO2 Lithium Batteries. Adv. Sci. 2017, 4, 1–9. 10.1002/advs.201600377 PMC5323859 28251055

[ref3] Porcarelli L.; Gerbaldi C.; Bella F.; Nair J. R. Super Soft All-Ethylene Oxide Polymer Electrolyte for Safe All-Solid Lithium Batteries. Sci. Rep. 2016, 6, 1–14. 26791572 10.1038/srep19892 PMC4726218

[ref4] Zhang J.; Zang X.; Wen H.; Dong T.; Chai J.; Li Y.; Chen B.; Zhao J.; Dong S.; Ma J.; Yue L. P.; Liu Z. H.; Guo X. X.; Cui G. L.; Chen L. Q. High-Voltage and Free-Standing Poly(Propylene Carbonate)/Li_6.75_La_3_Zr_1.75_Ta_0.25_O_12_ Composite Solid Electrolyte for Wide Temperature Range and Flexible Solid Lithium Ion Battery. J. Mater. Chem. A 2017, 5, 4940–4948.

[ref5] Kim J. G.; Son B.; Mukherjee S.; Schuppert N.; Bates A.; Kwon O.; Choi M. J.; Chung H. Y.; Park S. A Review of Lithium and Non-Lithium Based Solid State Batteries. J. Power Sources 2015, 282, 299–322.

[ref6] Zhang J.; Zhao J.; Yue L.; Wang Q.; Chai J.; Liu Z.; Zhou X.; Li H.; Guo Y.; Cui G.; Chen L. Q. Safety-Reinforced Poly(Propylene Carbonate)-Based All-Solid-State Polymer Electrolyte for Ambient-Temperature Solid Polymer Lithium Batteries. Adv. Energy Mater. 2015, 5, 1–10.26190957

[ref7] Liu W. Multilayer Composite Solid Electrolytes for Lithium Ion, 2016, Dissertation.

[ref8] Liang Y. F.; Xia Y.; Zhang S. Z.; Wang X. L.; Xia X. H.; Gu C. D.; Wu J. B.; Tu J. P. A Preeminent Gel Blending Polymer Electrolyte of Poly(Vinylidene Fluoride-Hexafluoropropylene) -Poly(Propylene Carbonate) for Solid-State Lithium Ion Batteries. Electrochim. Acta 2019, 296, 1064–1069.

[ref9] Zhang J.; Yue L.; Kong Q.; Liu Z.; Zhou X.; Zhang C.; Xu Q.; Zhang B.; Ding G.; Qin B.; Duan Y.; Wang J.; Yao G.; Cui G.; Chen L. Q. Sustainable, Heat-Resistant and Flame-Retardant Cellulose-Based Composite Separator for High-Performance Lithium Ion Battery. Sci. Rep. 2014, 4, 1–8. 10.1038/srep03935 PMC3909895 24488228

[ref10] Freitag K. M.; Kirchhain H.; Nilges T. Enhancement of Li Ion Conductivity by Electrospun Polymer Fibers and Direct Fabrication of Solvent-Free Separator Membranes for Li Ion Batteries. Inorg. Chem. 2017, 56, 2100–2107. 28150938 10.1021/acs.inorgchem.6b02781

[ref11] Yu H. F.; Kao S. Y.; Lu H. C.; Lin Y. F.; Feng H.; Pang H. W.; Vittal R.; Lin J. J.; Ho K. C. Electrospun Nanofibers Composed of Poly(Vinylidene Fluoride-Co-Hexafluoropropylene) and Poly(Oxyethylene)-Imide Imidazolium Tetrafluoroborate as Electrolytes for Solid-State Electrochromic Devices. Sol. Energy Mater. Sol. Cells 2018, 177, 32–43.

[ref12] Wang C.; Yang Y.; Liu X.; Zhong H.; Xu H.; Xu Z.; Shao H.; Ding F. Suppression of Lithium Dendrite Formation by Using LAGP-PEO (LiTFSI) Composite Solid Electrolyte and Lithium Metal Anode Modified by PEO (LiTFSI) in All-Solid-State Lithium Batteries. ACS Appl. Mater. Interfaces 2017, 9, 13694–13702. 28334524 10.1021/acsami.7b00336

[ref13] Reddy M. J.; Kumar J. S.; Subba Rao U. V.; Chu P. P. Structural and Ionic Conductivity of PEO Blend PEG Solid Polymer Electrolyte. Solid State Ionics 2006, 177, 253–256.

[ref14] Tenhaeff W. E.; Yu X.; Hong K.; Perry K. A.; Dudney N. J. Ionic Transport across Interfaces of Solid Glass and Polymer Electrolytes for Lithium Ion Batteries. J. Electrochem. Soc. 2011, 158, A1143–A1149.

[ref15] Gorecki W.; Jeannin M.; Belorizky E.; Roux C.; Armand M. Physical Properties of Solid Polymer Electrolyte PEO(LiTFSI) Complexes. J. Phys. Condens. Matter. 1995, 7, 6823–6832.

[ref16] Yu X. Y.; Xiao M.; Wang S. J.; Zhao Q. Q.; Meng Y. Z. Fabrication and Characterization of PEO/PPC Polymer Electrolyte for Lithium-Ion Battery. J. Appl. Polym. Sci. 2010, 115, 2718–2722.

[ref17] Edman L.; Doeff M. M.; Ferry A.; Kerr J.; De Jonghe L. C. Transport Properties of the Solid Polymer Electrolyte System P(EO)NLiTFSI. J. Phys. Chem. B 2000, 104, 3476–3480.

[ref18] Zhang H.; Liu C.; Zheng L.; Xu F.; Feng W.; Li H.; Huang X.; Armand M.; Nie J.; Zhou Z. Lithium Bis(Fluorosulfonyl)Imide/Poly(Ethylene Oxide) Polymer Electrolyte. Electrochim. Acta 2014, 133, 529–538.

[ref19] Zhu L.; Zhu P.; Yao S.; Shen X.; Tu F. High-Performance Solid PEO/PPC/LLTO-Nanowires Polymer Composite Electrolyte for Solid-State Lithium Battery. Int. J. Energy Res. 2019, 43, 4854–4866.

[ref20] Wang Z.; Gu H.; Wei Z.; Wang J.; Yao X.; Chen S. Preparation of New Composite Polymer Electrolyte for Long Cycling All-Solid-State Lithium Battery. Ionics 2019, 25, 907–916.

[ref21] Yue H.; Li J.; Wang Q.; Li C.; Zhang J.; Li Q.; Li X.; Zhang H.; Yang S. Sandwich-Like Poly(Propylene Carbonate)-Based Electrolyte for Ambient-Temperature Solid-State Lithium Ion Batteries. ACS Sustainable Chem. Eng. 2018, 6, 268–274.

[ref22] Ebadi M.; Marchiori C.; Mindemark J.; Brandell D.; Araujo C. M. Assessing Structure and Stability of Polymer/Lithium-Metal Interfaces from First-Principles Calculations. J. Mater. Chem. A 2019, 7, 8394–8404.

[ref23] Herbert E. G.; Tenhaeff W. E.; Dudney N. J.; Pharr G. M. Mechanical Characterization of LiPON Films Using Nanoindentation. Thin Solid Films 2011, 520, 413–418.

[ref24] Fleutot B.; Pecquenard B.; Martinez H.; Letellier M.; Levasseur A. Investigation of the Local Structure of LiPON Thin Films to Better Understand the Role of Nitrogen on Their Performance. Solid State Ionics 2011, 186, 29–36.

[ref25] Lacivita V.; Artrith N.; Ceder G. Structural and Compositional Factors That Control the Li-Ion Conductivity in LiPON Electrolytes. Chem. Mater. 2018, 30, 7077–7090.

[ref26] Wang B.; Kwak B. S.; Sales B. C.; Bates J. B. Ionic Conductivities and Structure of Lithium Phosphorus Oxynitride Glasses. J. Non-Cryst. Solids 1995, 183, 297–306.

[ref27] Schwöbel A.; Hausbrand R.; Jaegermann W. Interface Reactions between LiPON and Lithium Studied by In-Situ X-Ray Photoemission. Solid State Ionics 2015, 273, 51–54.

[ref28] Martha S. K.; Nanda J.; Kim Y.; Unocic R. R.; Pannala S.; Dudney N. J. Solid Electrolyte Coated High Voltage Layered-Layered Lithium-Rich Composite Cathode: Li_1.2_Mn_0.525_Ni_0.1_75Co _0.1_O_2_. J. Mater. Chem. A 2013, 1, 5587–5595.

[ref29] Park C. Bi-Layer Lithium Phosphorous Oxynitride/Aluminium Substituted Lithium Lanthanum Titanate as a Promising Solid Electrolyte for Long-Life Rechargeable Lithium – Oxygen Batteries. J. Mater. Chem. A 2015, 3, 22421–22431.

[ref30] Nimisha C. S.; Rao G. M.; Munichandraiah N.; Natarajan G.; Cameron D. C. Chemical and Microstructural Modifications in LiPON Thin Films Exposed to Atmospheric Humidity. Solid State Ionics 2011, 185, 47–51.

[ref31] Lin C. F.; Noked M.; Kozen A. C.; Liu C.; Zhao O.; Gregorczyk K.; Hu L.; Lee S. B.; Rubloff G. W. Solid Electrolyte Lithium Phosphous Oxynitride as a Protective Nanocladding Layer for 3D High-Capacity Conversion Electrodes. ACS Nano 2016, 10, 2693–2701. 26820038 10.1021/acsnano.5b07757

[ref32] Kim H. T.; Mun T.; Park C.; Wan S.; Young H. Characteristics of Lithium Phosphorous Oxynitride Thin Films Deposited by Metal-Organic Chemical Vapor Deposition Technique. J. Power Sources 2013, 244, 641–645.

[ref33] Teran A. A.; Tang M. H.; Mullin S. A.; Balsara N. P. Effect of Molecular Weight on Conductivity of Polymer Electrolytes. Solid State Ionics 2011, 203, 18–21.

[ref34] Su M. S.; Ahmad A.; Rahman M. Y. A. Ionic Conductivity Studies of 49% Poly(Methyl Methacrylate )-Grafted Natural Rubber-Based Solid Polymer Electrolytes. Ionics 2009, 15, 497–500.

[ref35] Choi C. H.; Cho W. I.; Cho B. W.; Kim H. S.; Yoon Y. S.; Tak Y. S. Radio-Frequency Magnetron Sputtering Power Effect on the Ionic Conductivities of LiPON Films. Electrochem. Solid State Lett. 2002, 5, 14–17.

[ref36] Verma M. L.; Sahu H. D. Study on Ionic Conductivity and Dielectric Properties of PEO-Based Solid Nanocomposite Polymer Electrolytes. Ionics 2017, 23, 2339–2350.

[ref37] Zhang J.; Yang J.; Dong T.; Zhang M.; Chai J.; Dong S.; Wu T.; Zhou X.; Cui G. Aliphatic Polycarbonate-Based Solid-State Polymer Electrolytes for Advanced Lithium Batteries: Advances and Perspective. Small 2018, 14, 1–16. 10.1002/smll.201800821 30073772

[ref38] Li X.; Wang Z.; Lin H.; Liu Y.; Min Y.; Pan F. Composite Electrolytes of Pyrrolidone-Derivatives-PEO Enable to Enhance Performance of All Solid State Lithium-Ion Batteries. Electrochim. Acta 2019, 293, 25–29.

[ref39] Boukamp B. A.; Huggins R. A. Lithium Ion Conductivity in Lithium Nitride. Phys. Lett. A 1976, 58, 231–233.

[ref40] Nazri G. Preparation, Structure and Ionic Conductivity of Lithium Phosphide. Solid State Ionics 1989, 34, 97–102.

