# Supplementary material for: Investigating the Effects of Lithium Phosphorous Oxynitride Coating on Blended Solid Polymer Electrolytes
Source: ACS Appl Mater Interfaces. 2020 Aug 13;12(36):40749–58. doi: 10.1021/acsami.0c09113 (PMC10905425; doi:10.1021/acsami.0c09113)
Supplement: Supplementary file 1 — am0c09113_si_001.pdf [file am0c09113_si_001.pdf]

Supporting Information for:

Investigating the Effects of Lithium Phosphorous Oxynitride Coating on Blended Solid Polymer Electrolytes

Jed LaCoste[a,b], Zhifei Li[a], Yun Xu[a], Zizhou He[b], Drew Matherne[b], Andriy Zakutayev*[a], Ling Fei*[a,b]

a. National Renewable Energy Laboratory, Materials Science Center, Golden, Colorado, 80401, United States
b. University of Louisiana Lafayette, Department of Chemical Engineering, Lafayette, Louisiana, 70504, United States
*Corresponding authors: ling.fei@louisiana.edu, andriy.zakutayev@nrel.gov

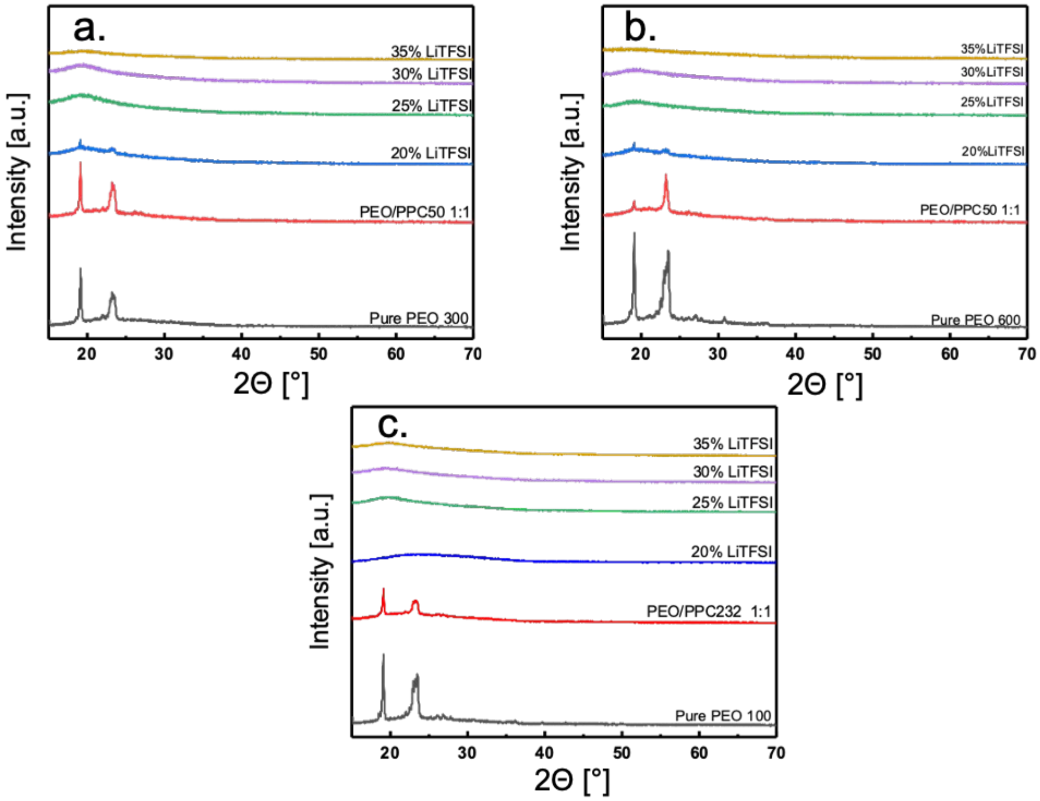

Figure S1: XRD patterns for a)PEO300, PEO300PPC50 (1:1) blend, and the resulting electrolyte blends with LiTFSI, b)PEO600, PEO600PPC50 (1:1) blend, and the resulting electrolyte blends with LiTFSI, and c) PEO100, PEO100PPC232 (1:1) blend, and the resulting electrolyte blends with LiTFSI.

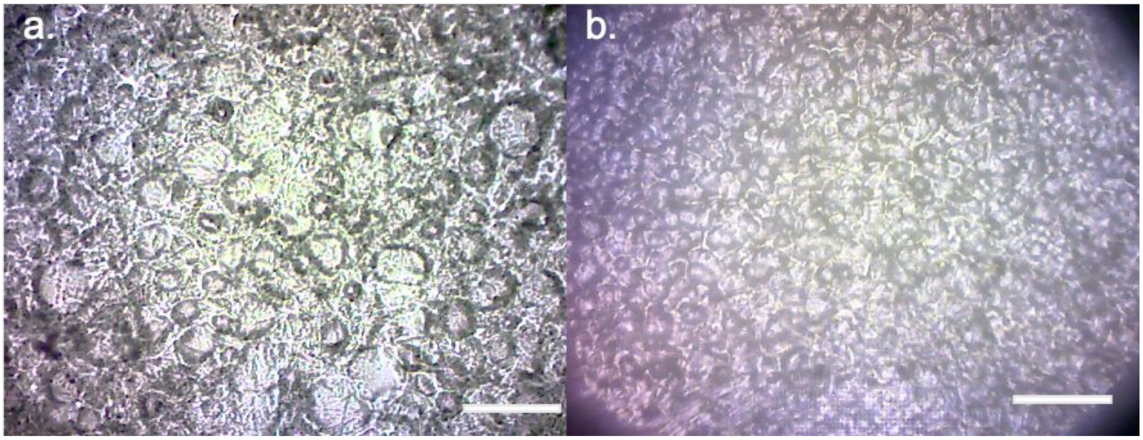

Figure S2: Optical Microscope Images of a) PEO100PPC232 (1:1), b) PEO100PPC232LiTFSI25, [Magnification 20x/ Scale Bar: 50 μm]

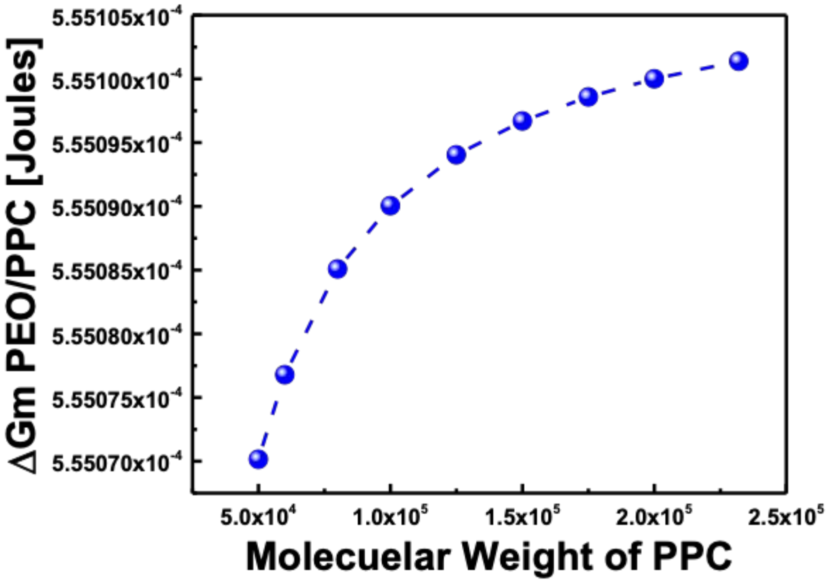

Figure S3: Calculated Gibb's free energy of mixing values for PEO100 with PPCx

Flory-Huggins equation calculation

To have an idea of the chain length influence in the blended polymer system, Flory-Huggins equation is used to determine polymer/polymer miscibility. From the Flory-Huggins equation the free energy of mixing, ΔG_m, is related to molecular weight and the interaction between polymer functional groups for polymer/liquid and polymer/polymer solutions. Different from ideal solution thermodynamics, Flory-Huggins solution theory accounts for the non-ideality of polymer blends by incorporating enthalpy of mixing in the following expression:[1]

$$\frac{\Delta G_m}{RT} = \frac{\phi_A}{m_A}\ln(\phi_A) + \frac{\phi_B}{m_B}\ln(\phi_B) + \phi_A\phi_B\chi_{AB}$$

Where ϕ_A and ϕ_B are the volume fractions of polymer A and B respectively, m_A and m_B are the molecular weight of polymers A and B respectively, and χ_{AB} is the interaction parameter for polymers A and B in solution. The chi interaction parameter is a unitless parameter that relates the functionality of the polymers to the miscibility and is determined by the solubility parameters of the polymers, δ. Symbolically, the value of chi is calculated through the following equation:[1]

$$\chi_{AB} = 0.34 + \frac{V_r}{RT}(\delta_A - \delta_B)^2$$

Where V_r is the reference volume, determined by the average molar volume of the polymer blend, and 0.34 is a correction factor related to the entropy of solution.[1,2] Determination of values is made possible through the use of tabulated group contributions and the expression relating the summation of molar attraction constants F* and the molar volume constant V*. Written in terms of polymer functional groups, i, the value of δ is related F* and V* through the following expression:

$$\delta = \frac{\sum_i F_i^*}{\sum_i V_i^*}$$

The individual group contributions are tabulated in table 11-1 from *Essentials of Polymer Science and Engineering* Edition 2 book and also copied here in Table S1 and S2 for PPC and PEO. The corresponding PPC and PEO functional groups classification are also shown below in Fig. S4 and S5, respectively.

Group	# of Groups	F* [(cal . cm^3)$^{0.5}$mol^{-1}]	V* (cm^3 mol^{-1})
$-CH_2-$	1	132	16.5
$-CH_3$	1	218	31.8
>CH-	1	23	1.9
-OCO-	1	298	19.6

Table S1: Tabulated group contributions for PPC

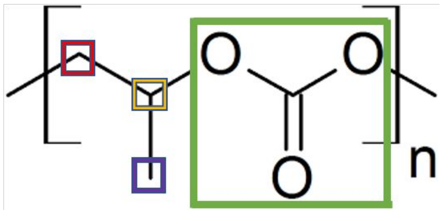

Figure S4: PPC's molecular structure with functional groups color coded: Red: $-CH_2-$, Purple: $-CH_3$, Yellow: >CH-, Green: -OCO-.

Group	# of Groups	F* [(cal . cm^3)$^{0.5}$mol^{-1}]	V* (cm^3 mol^{-1})
-CH_2-	2	132	16.5
-O-	1	95	5.1

Table S2: Tabulated group contributions for PEO.

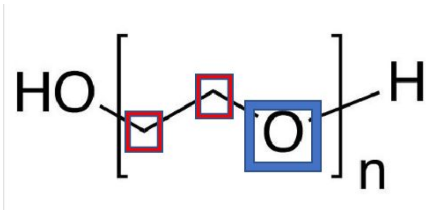

Figure S5: PPC's molecular structure with functional groups color coded: Red: -CH_2-, Blue: -O-.

From this, the δ values are 9.613 and 9.423 (cal. cm^{-3})$^{0.5}$ for PPC and PEO respectively resulting in a χ_{AB} value of 0.344. Values ϕ_A and ϕ_B are 0.647 and 0.353 respectively, determined by molar volume constants. The calculation result is plotted in Fig. S3. It can be seen that the change in free energy of mixing increases with increasing PPC molecular weight, making studying high molecular weight systems difficult due to higher degrees of immiscibility. Experimentally, in high molecular weight samples, extreme phase separation is noticed even with the addition of LiTFSI, agreeing with the exact trend we observed from the calculated plot. Therefore, high molecular weight of PPC indeed will result in poor miscibility which leads to poor electrolyte properties. PPC molecular weight less than 50K is very promising for further improving the system. It should also be noted that the role of the lithium salt is not included in this theoretical calculation. The lithium salt from our experimental result can promote miscibility but still not effective enough with high molecular weight PPC system.

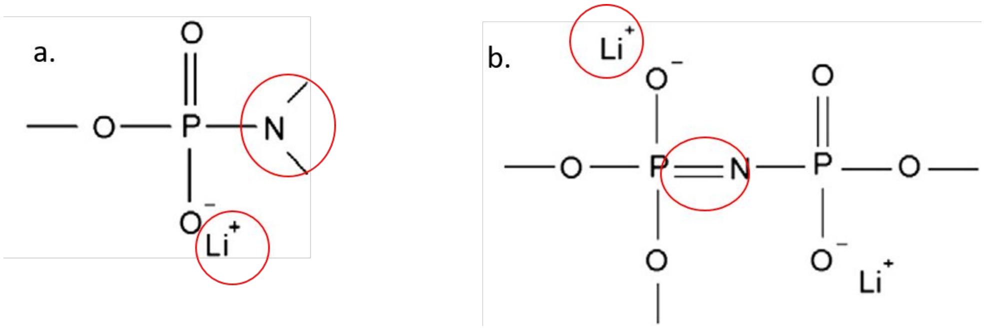

Figure S6: (a) the triple bond N and (b) double bond N in LiPON.

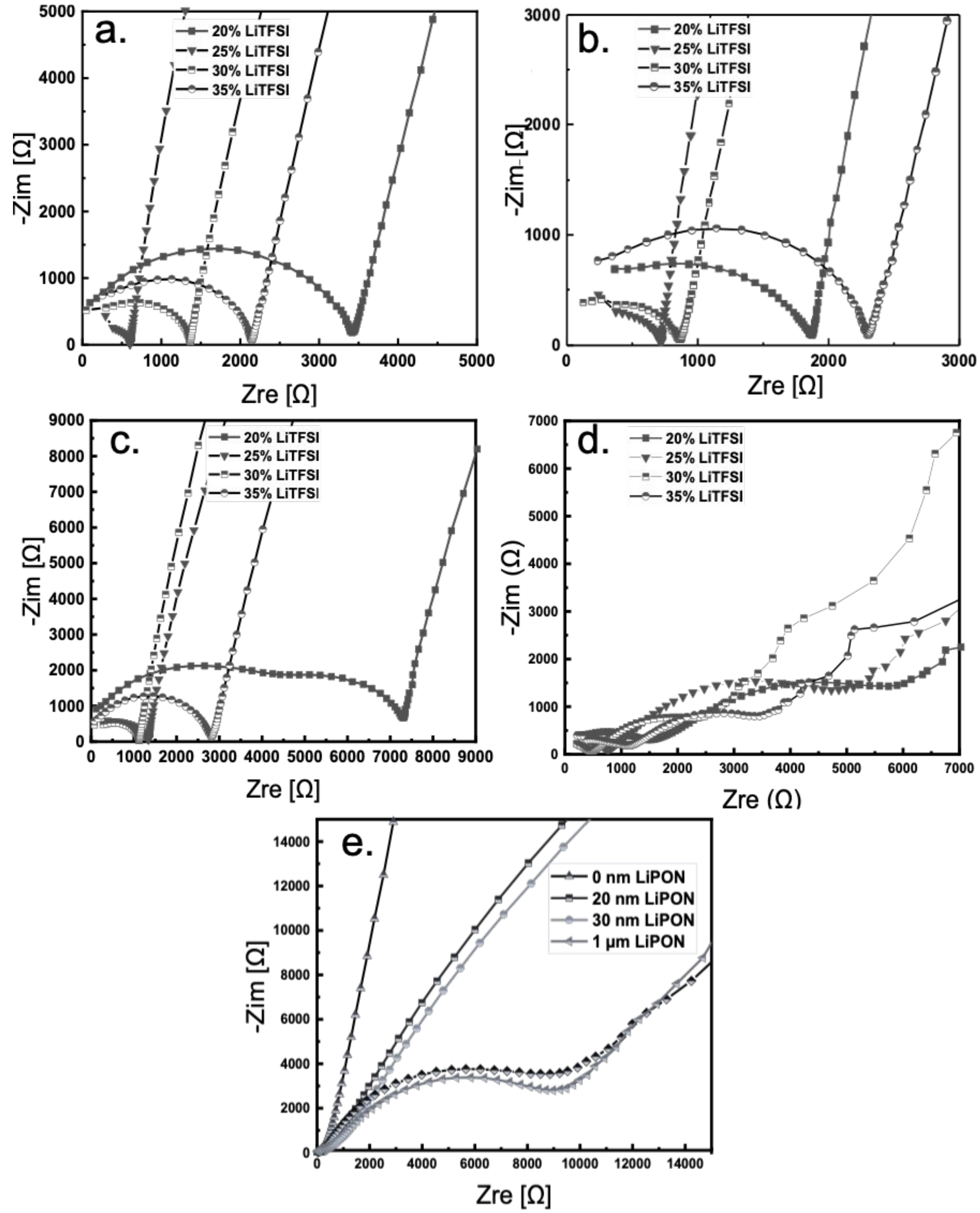

Figure S7: Impedance spectra for a) PEO100PPC50LiTFSIz, b) PEO300PPC50LiTFSIz, c) PEO600PPC50LiTFSIz, d) PEO100PPC232LiTFSIz, e) LiPON coated PEO100PPC50LiTFSI25

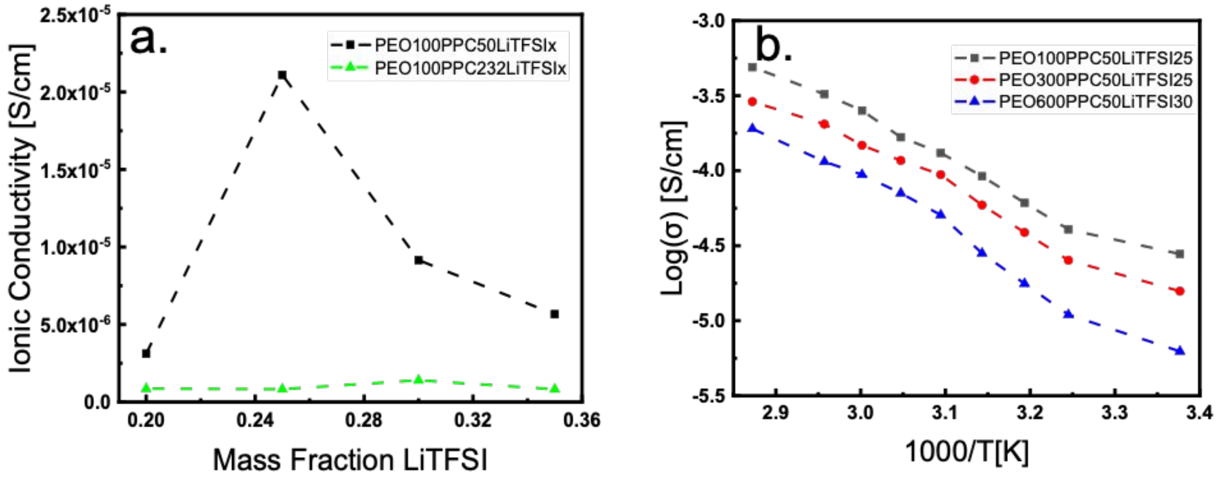

Figure S8: Ionic conductivity values for a) PEO100PPC50LiTFSIx and PEO100PPC232LiTFSIx and b) PEO100PPC50LiTFSI25, PEO300PPC50LiTFSI25, and PEO600PPC50LiTFSI30 over a range of temperatures (23-75 °C).

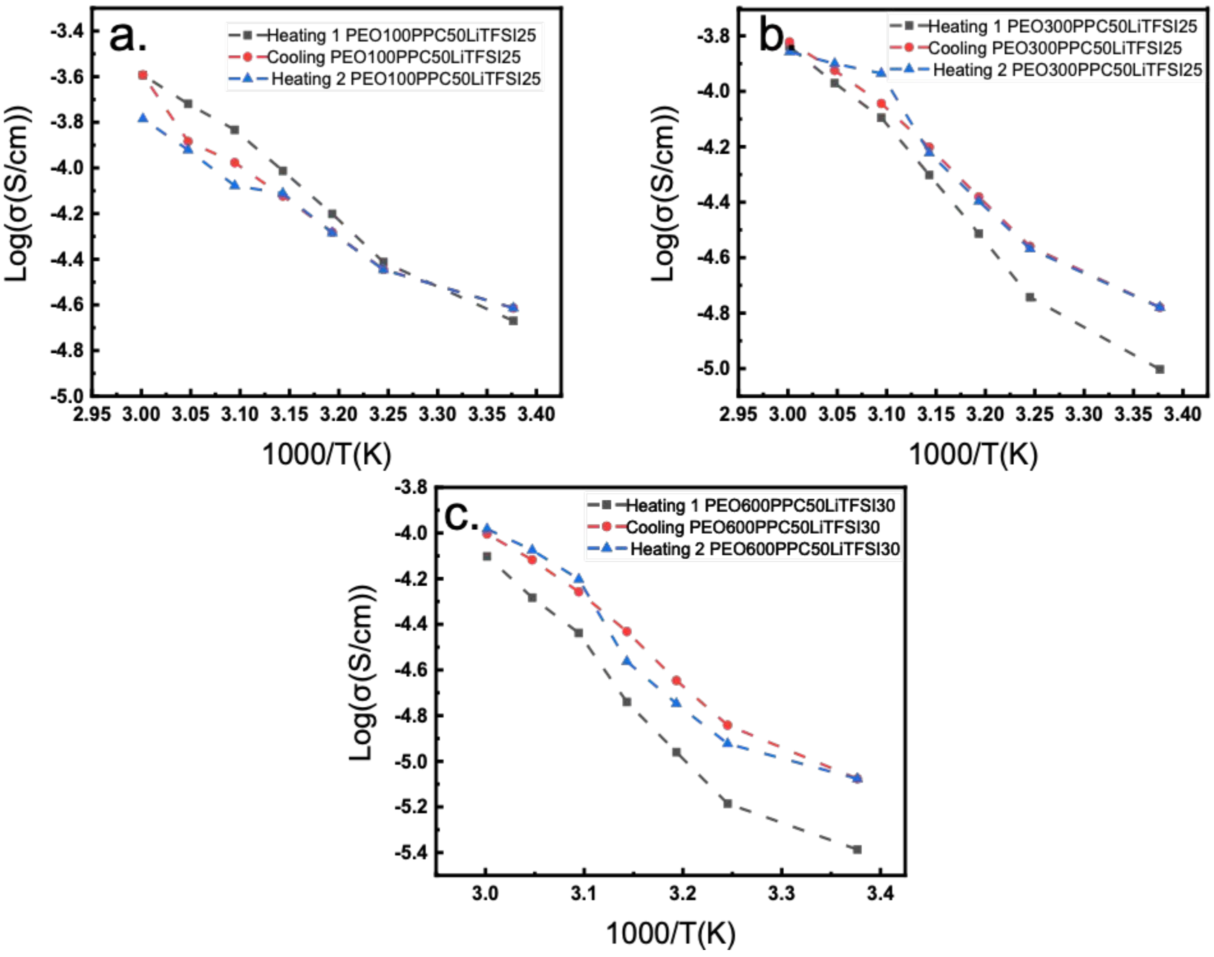

Figure S9: Thermal history dependence on ionic conductivity heating and cooling curves for a) PEO100PPC50LiTFSI25, b) PEO300PPC50LiTFSI25, and c) PEO600PPC50LiTFSI30.

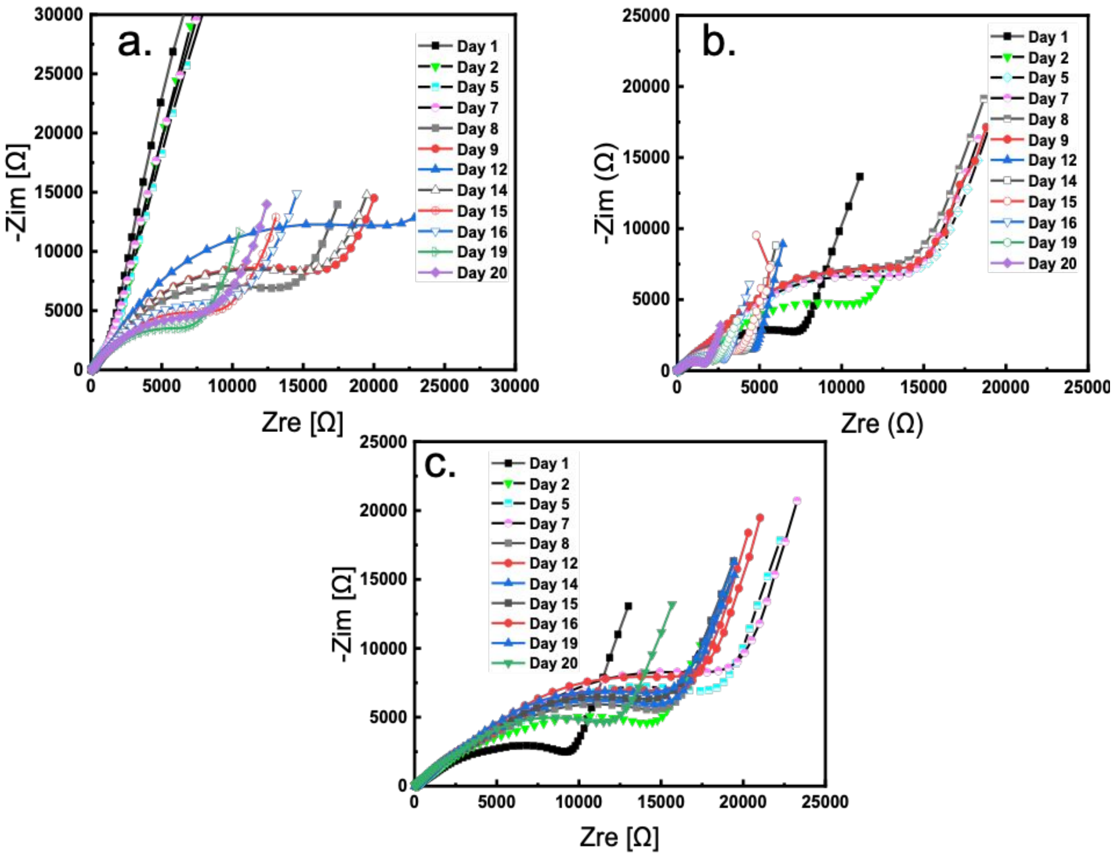

Figure S10: Electrochemical impedance spectroscopy of a) PEO100PPC50LiTFSI25 (BPE), BPE with 20 nm LiPON, and c) BPE with 30 nm LiPON to assess the effects of storage times of the electrolytes against lithium metal.

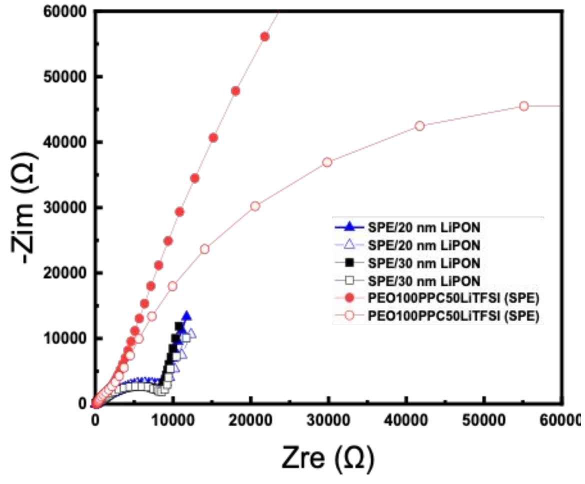

Figure S11: Impedance spectra of PEO100PPC50LiTFSI25 (SPE) and SPE coated with 20nm and 30nm LiPON taken against lithium metal and blocking electrode to evaluate the impedance response to polarization at 2V for 20 minutes (solid symbol before polarization, empty symbol after polarization).

References:

(1) Painter, P. C.; Coleman, M. M. Essentials of Polymer Science and Engineering; Chapter 11: Polymer Solutions and Blends. Lancaster, PA, 2009.

(2) Fried, J. R. *Polymer Science and Technology*, Chapter 3: Conformations, Solutions, and Molecular Weight. 3rd edition.; Pearson, 2014.
